# Enhancing SO_2_ and NO_2_ Gas Sensing Using ZnCdO_2_‑Based Porous Nanosheets: A DFT Perspective

**DOI:** 10.1021/acsomega.5c05129

**Published:** 2025-08-13

**Authors:** Warda Elaggoune, Nicolas F. Martins, Julio R. Sambrano, Yusuf Zuntu Abdullahi

**Affiliations:** † Laboratoire de Physique des Matériaux (L2PM), Faculté des mathématiques, de l’informatique et des sciences de la matière, Université 8 Mai 1945, BP 401, 24000 Guelma, Algeria; ‡ Modeling and Molecular Simulation Group, School of Sciences, 327031 São Paulo State University (UNESP), 17033-360 Bauru, SP, Brazil; § Department of Physics, 52943 Aydin Adnan Menderes University, Aydin 09010, Turkey; ∥ Department of Physics, Faculty of Science, Kaduna State University, PMB 2339, Kaduna 800283, Nigeria

## Abstract

The exceptional electronic properties, high surface area, and structural versatility of two-dimensional materials make them excellent candidates for gas-sensing applications. In this study, we propose novel biphenylene (b) and graphenylene (g) lattices of ZnCdO_2_ and explore their potential for detecting NO_2_ and SO_2_ gases via density functional theory calculations. The dynamic and thermal stability of b­(g)-ZnCdO_2_ monolayers is confirmed through phonon dispersion and ab initio molecular dynamics simulations. Both gases exhibit favorable adsorption on the monolayers, with significant charge transfer and electronic interaction. Notably, SO_2_ interaction on g-ZnCdO_2_ is characterized by weak chemisorption, supported by moderate adsorption energy, long-range interaction, and clear surface bonding, suggesting reusability under ambient conditions. Gas adsorption also induces substantial modulation in the work function, reinforcing the suitability of these monolayers for work-function-type sensing. In particular, the g-ZnCdO_2_+SO_2_ system shows an ultrafast recovery time at room temperature, with improved desorption kinetics at elevated temperatures. These insights position b­(g)-ZnCdO_2_ monolayers as promising platforms for efficient and reusable toxic gas sensors.

## Graphical abstract

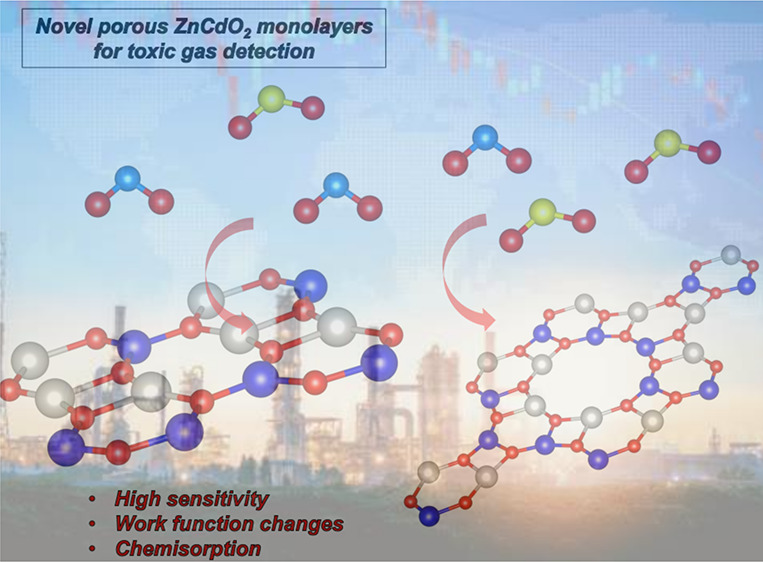

## Introduction

Carbon’s remarkable ability to adopt sp, sp^2^, and sp^3^ hybridizations has led to an extraordinary spectrum of low-dimensional allotropes, each possessing unique structural and electronic properties. Among these, novel topological carbon structures such as T-graphene,^1^ twin-graphene,^2^ penta-graphene,^3^ graphenylene,^4^ and biphenylene^5^ have garnered significant interest for their promising functionalities in modern energy harvesting and related applications. Biphenylene and graphenylene are particularly notable for their distinct lattice configurations—biphenylene featuring four-, six-, and eight-membered rings, and graphenylene characterized by a 4–6–12 topology—which endow them with exceptional functional versatility.^6,7^ Recent breakthroughs in their experimental synthesis, including biphenylene via HF-zipping dehydrogenation^8^ and graphenylene from the polymerization of 1,3,5-trihydroxybenzene precursors,^9^ have transformed these theoretical materials into tangible realities. Supported by rigorous density functional theory (DFT) studies, these topological carbon allotropes demonstrate immense promise across various fields, including catalysis,^10^ molecular membranes,^4^ gas sensing,^11^ energy storage,^12^ and optoelectronics.^13^

Despite their appealing attributes, biphenylene’s intrinsic metallic nature and graphenylene’s fixed bandgap pose limitations to their adaptability in semiconductor technologies requiring tunable electronic properties. This fundamental challenge has propelled research toward inorganic analogs, particularly within Group III, IV, and V semiconductors. Examples include boron nitride (BN),^14^ aluminum nitride (AlN),^15^ gallium nitride (GaN),^15^ indium nitride (InN),^16^ silicon carbide (SiC),^17^ germanium carbide (GeC),^18^ and silicon–germanium (SiGe)^19^ monolayers, alongside other related two-dimensional structures.^20–23^ However, oxide-based analogs of biphenylene and graphenylene topologies remain largely unexplored, representing a significant avenue for material innovation with potentially unique properties.

Among metal oxides, ZnO and CdO have garnered considerable attention due to their outstanding electronic properties, high surface reactivity, and oxygen-vacancy-driven adsorption mechanisms, making them highly suitable for advanced electronic and sensing applications.^24^ Hexagonal ZnO (hexa-ZnO) has been successfully synthesized in various nanostructures and widely applied in gas sensors, transparent electrodes, and UV photodetectors.^25,26^ Notable experimental advancements, such as the pulsed laser deposition of ZnO films^27^ and large-area ZnO monolayer synthesis via a graphene oxide template method,^28^ have further expanded their application scope, including efficient photocatalytic degradation demonstrated by ZnO nanosheets.^29^ Similarly, thin films of hexagonal CdO (hexa-CdO) have been synthesized through various methods^30,31^ and are extensively utilized in optical devices, leveraging their narrow direct bandgap, high conductivity, moderate electron mobility, and high carrier concentration.^32,33^

Building on these experimental and theoretical advancements, recent theoretical studies have explored biphenylene- and graphenylene-type structures of ZnO and CdO, hypothesizing that these novel forms might offer superior properties compared to their conventional hexagonal counterparts. Indeed, theoretical evidence suggests that biphenylene- and graphenylene-like ZnO and CdO monolayers are ultrawide-gap materials exhibiting excellent energetic, mechanical, dynamic, and thermal stability.^34^ Furthermore, investigations into their thickness-dependent bandgap variations have revealed a uniform decrease in bandgap as the number of XO-biphenylene and -graphenylene layers increases, further broadening their potential for next-generation applications.

A critical aspect for the practical deployment of these metal-oxide-based biphenylene and graphenylene structures is their stability and responsiveness to environmental gas molecules. Their unique atomic arrangements and nanoscale thickness mean that interactions with common atmospheric species, such as nitrogen, oxygen, and sulfur, can significantly influence their structural integrity and electronic properties. In particular, sulfur dioxide (SO_2_) and nitrogen dioxide (NO_2_) are highly hazardous air pollutants derived from fossil fuel combustion. The precise and rapid detection of these toxic gases is paramount not only for environmental monitoring but also for safeguarding public health and mitigating safety risks.

Although numerous two-dimensional materials such as graphene and its derivatives,^35–37^ MoS_2_,^38,39^ h-BN,^40^ ZnO,^41,42^ and CdO^43^ have been explored for gas sensing, each presents inherent limitations. Graphene offers exceptional mobility but lacks a bandgap, requiring external functionalization to achieve selectivity.^35^ MoS_2_, while possessing a suitable bandgap, suffers from modest sensitivity and carrier mobility.^38^ ZnO and CdO remain popular due to their high surface reactivity, but ZnO-based sensors typically demand high operating temperatures (above 300 K),^41^ and CdO often faces stability issues under ambient conditions.^43^ Even recent theoretical studies of hexagonal ZnCdO_2_ suggest improvements in photocatalytic properties but leave its sensing capabilities largely unexamined.^44^ In this context, biphenylene- and graphenylene-based ZnCdO_2_ monolayers are anticipated to surpass these limitations by combining topological advantages, ultrawide bandgaps, and hybrid electronic characteristics in a single platform. Their novel atomic arrangements may enable enhanced surface adsorption and selective charge transfer—two key factors in the design of next-generation gas sensors. Although these topological ZnCdO_2_ structures have not yet been experimentally synthesized, the feasibility of such 2D monolayers is supported by existing methods used to fabricate related oxides such as the synthesized thin films ZnCdO^45^ and Zn_1–*x*_Cd_*x*_O^46^ and nanotetrapods ZnCdO.^47^ Techniques such as atomic layer deposition (ALD), molecular beam epitaxy (MBE), and pulsed laser deposition (PLD) have successfully enabled the growth of ultrathin ZnO, CdO, and even heterostructured films with controlled composition and crystallinity.^48–52^ These approaches could be adapted to realize the biphenylene and graphenylene architectures through substrate templating or stepwise oxide growth. Thus, the synthesis of b-ZnCdO_2_ and g-ZnCdO_2_ may be achievable using current deposition technologies, opening the door to experimental validation of their predicted sensing performance.

Although several studies have explored the adsorption of NO_2_ and SO_2_ on biphenylene- and graphenylene-type ZnO and CdO monolayers, existing research remains limited to these binary oxide systems. To date, no investigations have addressed the gas-sensing performance of a hybrid ZnCdO_2_ composition integrated within such topological frameworks. This omission is significant given the complementary chemical and electronic features of Zn and Cd oxides, which could synergistically enhance the sensor response. Motivated by this gap, the present study provides a detailed theoretical investigation into the interaction dynamics of NO_2_ and SO_2_ with biphenylene-ZnCdO_2_ (b-ZnCdO_2_) and graphenylene-ZnCdO_2_ (g-ZnCdO_2_) monolayers. By analyzing adsorption energies, charge transfer, electronic structure, and work function modulation, we aim to assess the potential of these novel monolayers as high-performance platforms for toxic gas detection. Our findings are expected to offer valuable insight into the design of next-generation 2D gas sensors based on chemically versatile oxide frameworks.

## Computational Setup

In this study, density functional theory (DFT) calculations were performed using the Vienna Ab initio Simulation Package (VASP).^53–56^ Electron–ion interactions were described by the projector-augmented wave (PAW) method,^57^ and exchange-correlation effects were accounted for through the Perdew–Burke–Ernzerhof (PBE) functional within the Generalized Gradient Approximation (GGA) framework.^58^ Structural optimization employed Γ-centered *k*-point grids of 3 × 3 × 1 and 6 × 3 × 1 for the biphenylene and graphenylene atomic structures, respectively, followed by a denser *k*-mesh for accurate electronic property calculations. The self-consistent field (SCF) cycle was converged to an energy threshold of 1 × 10^–5^ eV per atom, while the maximum atomic force tolerance was set to 0.001 Ha/Å. To suppress artificial interactions from periodic boundary conditions, an 18 Å vacuum layer was introduced along the *z*-direction. A plane-wave cutoff energy of 500 eV was adopted to ensure calculation accuracy. van der Waals (vdW) interactions were incorporated using Grimme’s DFT-D3 correction scheme.^59^

The dynamical stability of the studied monolayers was examined via phonon dispersion calculations, performed using the supercell approach implemented in the PHONOPY package.^60^ These calculations provided insights into the phonon band structure and the atom-projected density of states (PDOS). Thermal stability was evaluated by performing finite-temperature ab initio molecular dynamics (AIMD) simulations at 300 K for 5000 time steps.^61^ The mechanical stability of the system was further verified by ensuring compliance with the Born–Huang criteria.^62^

For a more precise description of electronic properties, band structure calculations were refined using the Heyd–Scuseria–Ernzerhof (HSE06) screened hybrid functional.^63^ Charge transfer and bonding characteristics were analyzed using the Electron Localization Function (ELF) and Bader charge method,^64^ providing a detailed understanding of the adsorption interactions on the monolayer surfaces.

## Results and Discussion

### Geometrical Structures of Bare b-ZnCdO_2_ and g-ZnCdO_2_ Monolayers

Figure 1a,b presents the optimized atomic structures of the pristine b-ZnCdO_2_ and g-ZnCdO_2_ monolayers. The b-ZnCdO_2_ monolayer features a periodic arrangement of 4-, 6-, and 8-membered rings, forming a tetra-hexa-octa framework that departs from the conventional hexagonal symmetry observed in graphene. In contrast, the g-ZnCdO_2_ monolayer exhibits a dodecagonal network, where 12-membered rings are systematically enclosed by 4- and 6-membered rings, contributing to its extended lattice structure. Notably, each cavity within these frameworks maintains a nondangling bond, with dimensions determined by the atomic composition of the unit cell. Structurally, each unit cell comprises three Zn, three Cd, and six O atoms, each coordinated with three neighboring atoms, which ensures lattice stability.

**Figure 1:**
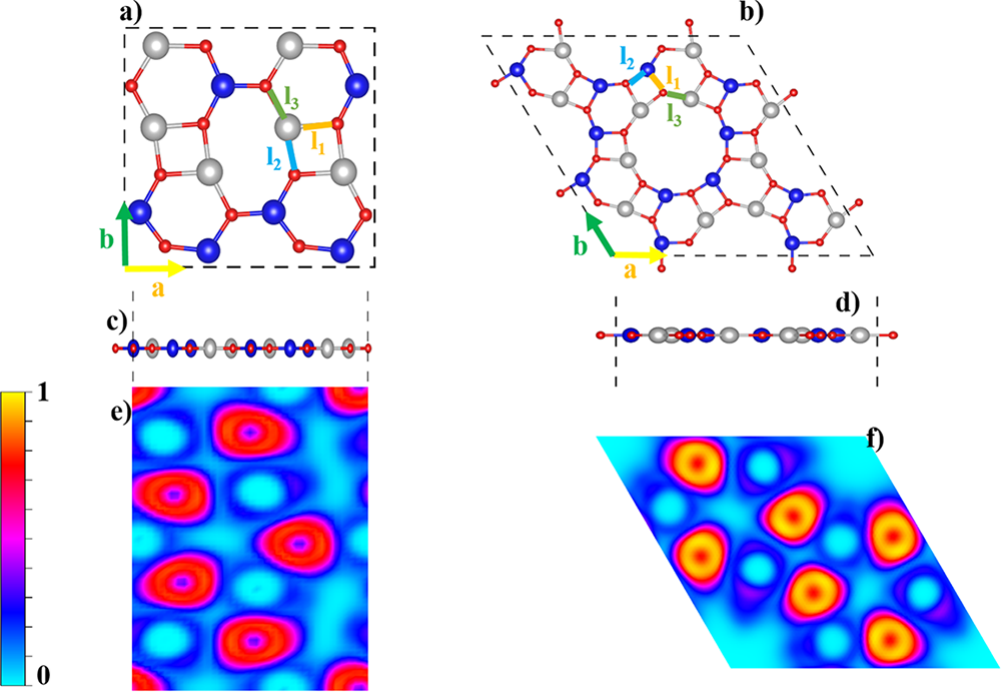
(a, b) Top views and (c, d) side views of the optimized 2 × 2 × 1 supercell structures of b-ZnCdO_2_ (left panels) and g-ZnCdO_2_ (right panels). The atomic species are color-coded as follows: blue for Zn, gray for Cd, and red for O atoms. (e, f) Electron Localization Function (ELF) maps for b-ZnCdO_2_ and g-ZnCdO_2_ monolayers, respectively. High ELF values (red to yellow) indicate strong electron localization, particularly around oxygen atoms, reflecting the ionic character of the bonds.

Figure 1c,d provides a side view of the optimized monolayers, revealing that both b-ZnCdO_2_ and g-ZnCdO_2_ preserve the intrinsic planar geometry characteristic of their carbon-based biphenylene and graphenylene prototypes, exhibiting no significant buckling distortions after optimization. The lattice constants obtained after geometric optimization, as listed in Table 1, are *a* = 6.07 Å, *b* = 10.72 Å for b-ZnCdO_2_, and *a* = 9.38 Å for g-ZnCdO_2_. These values are consistent with previously reported analogs, such as b-ZnMgO_2_ (*a* = 5.76 Å)^65^ and g-ZnMgO_2_ (*a* = 8.91 Å),^23^ and are significantly larger than that of hexagonal ZnCdO_2_ (h-ZnCdO_2_), which has a smaller lattice constant of 3.61 Å.^44^ The bond lengths defining various ring connections within the monolayers—namely *l*_1_ (shared between hexagons and squares), *l*_2_ (shared between squares and octa-/dodecagons), and *l*_3_ (shared between hexagons and octa-/dodecagons)—range from 2.06 to 2.25 Å for b-ZnCdO_2_ and 1.88 to 2.08 Å for g-ZnCdO_2_, as depicted in Figure 1a,b and summarized in Table 1. These values underscore the structural robustness of the monolayers. The b-ZnCdO_2_ monolayer, with its smaller ring sizes and stronger atomic interactions, forms a more compact and tightly bonded framework, resulting in reduced lattice dimensions. Conversely, the g-ZnCdO_2_ monolayer, characterized by 12-membered rings and larger cavities, exhibits a more open and flexible architecture, leading to increased lattice parameters. Compared to their hexa-counterpart (*r*_Zn_ = *r*_Cd_ = 2.09 Å for h-ZnCdO_2_),^44^ the Zn/Cd–O bond lengths in b-ZnCdO_2_ and g-ZnCdO_2_ remain close, with deviations of approximately 0.03 and 0.05 Å, respectively, based on the *l*_3_ bond shared between hexagonal and octagonal/dodecagonal rings. This slight elongation is attributed to the expanded lattice parameters in the b­(g)-frameworks, which accommodate larger polygonal rings.

**Table 1:** Lattice Constants (*a* and *b* in (Å)), Bond Lengths (*l*_1_, *l*_2_, and *l*_3_ in (Å)), Average Bader Charge Transfer (*Q* in (e^–^) for Different Atoms), Cohesive Energy (*E*_coh_ in (eV/atom)), Formation Energy (*E*_for_ in (eV/atom)), Young’s Modulus (*Y*_*a*_ and *Y_b_* in (N/m)), Poisson’s Ratio (ν_*a*_ and ν_*b*_), and Band Gap with Both PBE and HSE Methods of the b-ZnCdO_2_ and g-ZnCdO_2_ Monolayers

system	b-ZnCdO_2_	g-ZnCdO_2_
*a*/*b*	6.07/10.72	9.38
*l*_1_	2.25	2.02
*l*_2_	2.07	1.88
*l*_3_	2.06	2.04
*Q*_Zn_	1.19	1.19
*Q*_Cd_	1.10	1.10
*Q*_O_	–1.15	–1.15
*E*_coh_	3.09	3.03
*E*_for_	–0.86	–0.81
*Y_a_*/*Y_b_*	21.24/48.94	35.41
ν_*a*_/ν_*b*_	0.46/1.02	0.60
*E*_g_ (PBE/HSE)	1.29/3.06	1.80/3.13

To better understand the bonding nature in the b- and g-ZnCdO_2_ monolayers, we examined the Electron Localization Function (ELF). As shown in Figure 1e,f, regions with high ELF values (yellow to red) are mainly found around the oxygen atoms, indicating strong electron localization. This is due to the large difference in electronegativity between Zn (1.65), Cd (1.69), and O (3.44), which causes electrons to be strongly attracted to oxygen atoms. These localized regions reflect the ionic character of the bonding, which becomes more evident when moving from the biphenyl-like to the graphenyl-like structure. In contrast, lower ELF values (blue to cyan) appear between the atoms, suggesting some electron sharing and indicating a polar-covalent nature in the bonding framework.

The Bader charge analysis (Table 1) further confirms substantial charge transfer, with Zn and Cd atoms acting as electron donors and oxygen atoms as acceptors. The net charges calculated on Zn and Cd (∼+1.15*e*) are slightly lower than the expected +2 oxidation state, suggesting a degree of charge delocalization. This aligns with the ELF findings, reinforcing the presence of a strong ionic component along with a minor polar covalent contribution in ZnCdO_2_ monolayers. These results are consistent with previous studies on ZnMgO_2_ monolayers, where an average net charge of 1.40*e* was reported.^23^ Interestingly, despite the structural differences and the improved bonding characteristics, the total amount of charge transferred is nearly identical in both configurations, suggesting that the underlying charge transfer mechanism is largely unchanged.

The structural stability and synthetic feasibility of the ZnCdO_2_ monolayers were systematically examined by evaluating the cohesive energy (*E*_coh_) and the formation energy (*E*_f_). These quantities are computed using the following expressions:

$$E_{\mathrm{coh}}=\frac{3E_{\mathrm{Zn}}+3E_{\mathrm{Cd}}+6E_{O}-E_{{\mathrm{ZnCdO}}_{2}}}{12}$$ 1

$$E_{f}=\frac{E_{{\mathrm{ZnCdO}}_{2}}-3\mu \mathrm{Zn}-3\mu_{\mathrm{Cd}}-6\mu_{O}}{12}$$ 2

where *E*_ZnCdO_2__ is the total energy of the monolayer, while *E*_Zn_, *E*_Cd_, and *E*_O_ represent the total energies of isolated Zn, Cd, and O atoms, respectively. The chemical potentials μ_Zn_, μ_Cd_, and μ_O_ correspond to their most stable elemental phases, with Zn and Cd crystallizing in a hexagonal *P*_63_/*mmc* structure and O in a monoclinic C_2_/m configuration. Table 1 summarizes the results obtained, where the cohesive energy values for b-ZnCdO_2_ and g-ZnCdO_2_ are calculated to be 3.09 and 3.03 eV/atom, respectively. These values suggest a strong atomic framework and are comparable to those reported for ZnMgS_2_ (3.16 eV/atom),^23^ silicene (3.61 eV/atom),^66^ and experimentally realized germanene (3.74 eV/atom).^67^ Additionally, the negative formation energies confirm the thermodynamic stability of both monolayers. Interestingly, their magnitudes are comparable to that of g-BSb (−0.86 eV/atom),^68^ while exceeding those of purely carbon-based biphenylene (−0.49 eV/atom) and graphenylene (−0.62 eV/atom).^68^

The mechanical stability of the ZnCdO_2_ monolayers was assessed by computing their elastic constants (*C*_*ij*_) using the strain–stress method.^69,70^ The obtained values are *C*_11_ = 43.61 N/m, *C*_22_ = 87.58 N/m, *C*_12_ = 44.25 N/m, and *C*_66_ = 14.17 N/m for b-ZnCdO_2_; and *C*_11_ = 54.83 N/m, *C*_12_ = 32.64 N/m, and *C*_66_ = 11.10 N/m for g-ZnCdO_2_. These values satisfy the stability criteria for rectangular and hexagonal structures, respectively.^62^

To gain deeper insights into their mechanical behavior, Young’s modulus (*Y*) and Poisson’s ratio (ν) were analyzed, as presented in Table 1. The b-ZnCdO_2_ monolayer exhibits pronounced anisotropy due to its rectangular symmetry, with distinct stiffness values along the *a* and *b* directions. In contrast, g-ZnCdO_2_, with its hexagonal symmetry, maintains isotropic mechanical properties. The Young’s modulus values indicate that g-ZnCdO_2_ possesses an intermediate stiffness, falling between the two main axes of b-ZnCdO_2_.

Poisson’s ratio further emphasizes the anisotropic nature of b-ZnCdO_2_, where ν_*b*_ = 1.02 suggests a significant lateral contraction along the *b*-axis, hinting at improved flexibility and ductility. Conversely, the *a*-axis (ν_*a*_ = 0.46) demonstrates reduced lateral deformation. Meanwhile, g-ZnCdO_2_ exhibits a more balanced mechanical response, making it mechanically resistant to external stress. In comparison, the obtained *Y*(ν) values agree well with those reported for g-SiGe (*Y* = 23.6 N/m, ν = 0.47)^19^ and b-BSb (*Y* = 27.01 N/m, ν = 0.45).^68^

To further evaluate the stability of the ZnCdO_2_ monolayers, we conducted phonon dispersion calculations and ab initio molecular dynamics (AIMD) simulations at room temperature (300 K). These analyses confirm the dynamical and thermal stability of the systems under ambient conditions. As shown in Figure 2a,b, the phonon spectra display no imaginary frequencies along the high-symmetry paths in the first Brillouin zone, indicating that both monolayers are dynamically stable. In the phonon dispersion curves, the in-plane longitudinal acoustic (LA) and transverse acoustic (TA) modes exhibit linear behavior. In contrast, the out-of-plane transverse acoustic (ZA) mode shows the expected quadratic dispersion near the Γ-point. The maximum phonon frequencies for both b-ZnCdO_2_ and g-ZnCdO_2_ exceed 750 cm^–1^, suggesting strong chemical bonding within the structures. This observation is consistent with the previously discussed structural characteristics and is comparable to those found in other well-known 2D materials. The projected phonon density of states (PDOS), shown in the right panel of Figure 2, offers further insight into the vibrational behavior. As expected from the mass-dependent nature of vibrational modes, lighter atoms contribute to higher-frequency vibrations. Thus, oxygen atoms dominate the high-frequency region, while the heavier Zn and Cd atoms contribute mainly to the lower-frequency modes. Notably, Zn atoms play a significant role in the acoustic modes, underlining their influence on the lattice dynamics.

**Figure 2:**
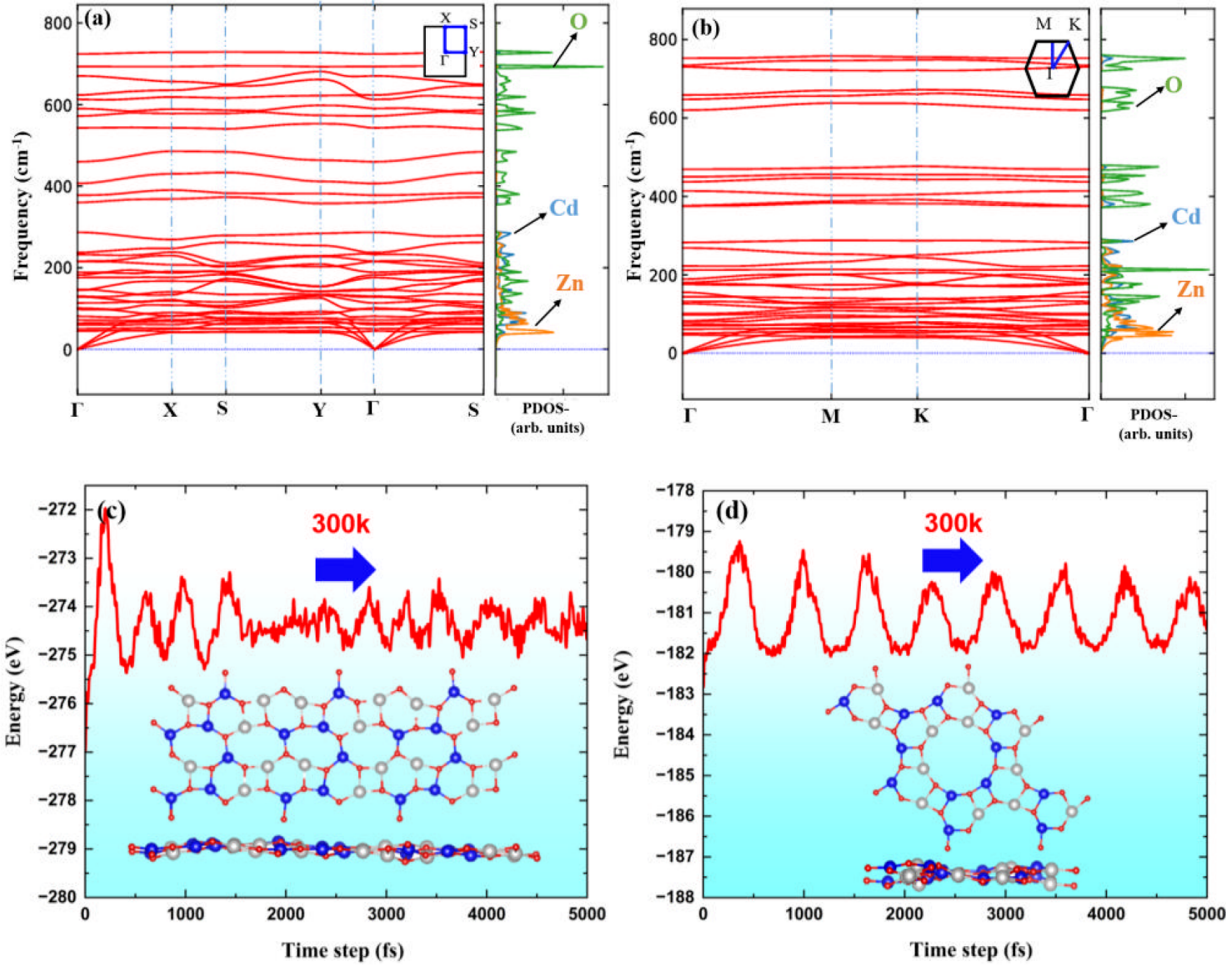
(a, b) Phonon dispersion relations (left panels) and the corresponding atom-projected phonon density of states (PDOS) (right panels) for b-ZnCdO_2_ and g-ZnCdO_2_ monolayers, respectively. The absence of imaginary frequencies across the Brillouin zone confirms dynamical stability. In the PDOS plots, contributions from different atoms (Zn, Cd, and O) are resolved, highlighting the vibrational role of each species. (c, d) Total energy evolution profiles during ab initio molecular dynamics (AIMD) simulations at 300 K for b-ZnCdO_2_ and g-ZnCdO_2_, respectively. Insets display the final top and side view snapshots of the relaxed atomic structures after the simulation, confirming thermal stability and structural integrity.

Figure 2c,d presents the total energy oscillations as a function of time at 300 K, providing insight into the thermal stability of the b-ZnCdO_2_ and g-ZnCdO_2_ monolayers. Inset snapshots depict the top and side views of these structures after 5 ps. The total energy remains stable throughout the simulation, fluctuating around the equilibrium state without significant atomic displacement. However, minor buckling is observed, particularly in the g-type structure. Also, minor changes in bond lengths can be observed. For instance, the inter-ring bonds in b-ZnCdO_2_ (*l*_2_ and *l*_3_) vary slightly (from 2.06/2.07 to 2.08 Å), while a more significant distortion is seen in the intraring Cd–O bond, which decreases from 2.25 to 2.17 Å. This behavior can be attributed to an increase in the buckling height. A similar trend is observed in the graphenylene-like lattice, with bond length changes from 2.02 to 1.99 Å (*l*_1_), 1.88 to 1.84 Å (*l*_2_), and 2.04 to 2.07 Å (*l*_3_). On the other hand, the pore diameters were modified by 0.17 and 0.22 Å for the b-ZnCdO_2_ and g-ZnCdO_2_ monolayers, respectively.

### The Electronic Properties of Bare b-ZnCdO_2_ and g-ZnCdO_2_ Monolayers

The electronic band structure and density of states (DOS) of the b-ZnCdO_2_ and g-ZnCdO_2_ monolayers are presented in Figure 3. The upper panel illustrates the band structures computed using the PBE functional, while the lower panel corresponds to the hybrid HSE functional results. The PBE-calculated DOS is embedded within the respective band structures as a reference.

**Figure 3:**
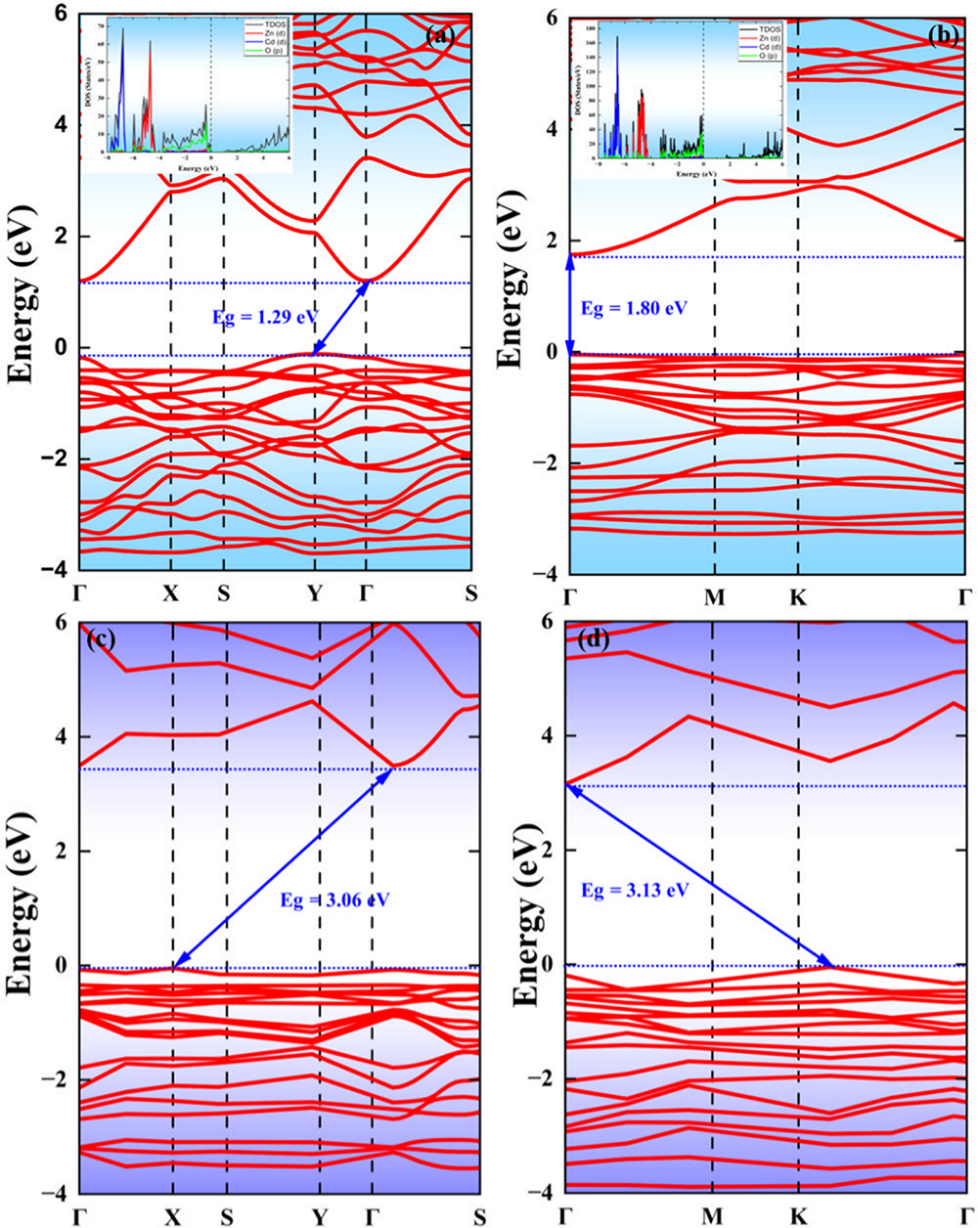
Band structure of b-ZnCdO_2_ and g-ZnCdO_2_ monolayers calculated using the PBE method (a, b) and the HSE method (c, d). The snapshots in (a and b) depict the corresponding total density of states (TDOS) and projected density of states (PDOS) obtained using the PBE method.

Both monolayers exhibit semiconducting behavior. Specifically, b-ZnCdO_2_ displays an indirect bandgap of 1.29 eV, with its valence band maximum (VBM) and conduction band minimum (CBM) located at Y and Γ, respectively. In contrast, g-ZnCdO_2_ possesses a direct bandgap of 1.80 eV, where both VBM and CBM are situated at Γ.

Upon employing the HSE functional, a significant widening of the bandgap is observed, with an increase of approximately 1.77 eV for b-ZnCdO_2_ and 1.33 eV for g-ZnCdO_2_. Additionally, the band extrema shift: for b-ZnCdO_2_, the VBM is at X and the CBM is located between Γ and S. Notably, g-ZnCdO_2_ undergoes a transition from a direct to an indirect bandgap, with a relocalization of its band extrema.

Examining the DOS profiles, the total density of states (TDOS) is predominantly governed by the Zn-d and Cd-d orbitals in the valence region, exhibiting pronounced peaks in the energy range of −8 to −4 eV. This signifies a strong hybridization between these metal d-states. The O-p orbitals contribute significantly near the VBM, suggesting their pivotal role in bonding interactions. In the conduction region, the DOS is relatively lower, with minor orbital contributions from all atomic species, affirming the presence of a well-defined bandgap. Notably, g-ZnCdO_2_ exhibits a higher DOS intensity compared to b-ZnCdO_2_, suggesting an enhanced orbital overlap in the g-type structure.

### Adsorption Properties of b­(g)-ZnCdO_2_ Monolayers for NO_2_ and SO_2_ Detection

Initially, the adsorption of NO_2_ and SO_2_ was examined at various available sites on the respective b­(g)-ZnCdO_2_ monolayers. Figure 4 illustrates the potential adsorption sites, including hollow (H1 → H3), atomic (A1 → A3), and bridge (B1 → B3) configurations on the analyzed sensing materials.

**Figure 4:**
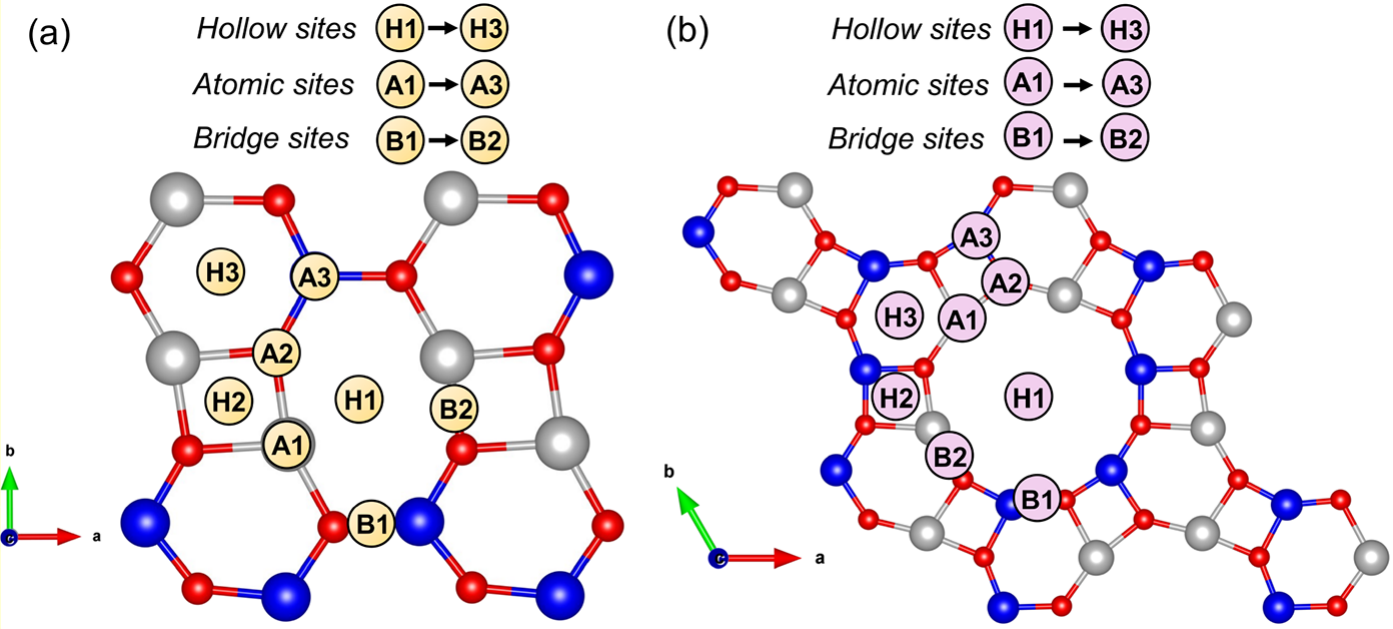
Top views of the (a) b-ZnCdO_2_ and (b) g-ZnCdO_2_ monolayers showing the available adsorption sites for potential adsorbates. Specific adsorption positions—such as atop metal atoms (Zn or Cd), atop O atoms, bridge sites, and hollow sites—are marked to illustrate the distinct local environments considered for adsorption energy calculations.

To understand the binding strength of the gas interaction on b­(g)-ZnCdO_2_ structures, the adsorption energy (*E*_ads_) was calculated using the following formula:

$$E_{\mathrm{ads}}=E_{{(\mathrm{b(g)‐ZnCdO}}_{2}+\mathrm{gas})}-E_{{(\mathrm{b(g)‐ZnCdO}}_{2})}-E_{\left(\mathrm{gas}\right)}$$ 3

where *E*_b(g)‑ZnCdO_2_+gas_, *E*_b(g)‑ZnCdO_2__, and *E*_gas_ represent the total energies of the b­(g)-ZnCdO_2_-gas system, the pristine b­(g)-ZnCdO_2_ monolayer, and the isolated optimized gas (NO_2_ and SO_2_). Table 2 presents the main adsorption parameters for each adsorptive system, including the most favorable site.

**Table 2:** Adsorption Energies (*E*_ads_), Minimum Interaction Distances (*d*), and Bader Charges (*Q*) for NO_2_ and SO_2_ on b­(g)-ZnCdO_2_ Structures

system	gas	stable site	*E*_ads_ (eV)	*d* (Å)	*Q* (*e*)
b-ZnCdO_2_	NO_2_	H1	–1.63	2.72	–0.22
	SO_2_	A3	–1.45	2.04	–0.17
g-ZnCdO_2_	NO_2_	H1	–1.88	2.58	–0.44
	SO_2_	H1	–0.55	2.99	–0.12

Both NO_2_ molecules preferentially adsorb at the H1 site of the b­(g)-ZnCdO_2_ monolayers, located at the center of the octagonal (dodecagonal) pores. For SO_2_, this preference is observed only on the g-ZnCdO_2_ monolayer, whereas on b-ZnCdO_2_, SO_2_ binds more stably at the A3 site. Figure 5 illustrates the side and top views of all optimized configurations. From an energetic standpoint, all systems exhibit thermodynamically stable adsorption, as evidenced by their significantly negative adsorption energies, confirming the strong affinity of the b­(g)-ZnCdO_2_ surfaces toward toxic gas molecules. Specifically, the computed *E*_ads_ values are −1.63 (NO_2_) and −1.45 eV (SO_2_) for b-ZnCdO_2_, and −1.88 (NO_2_) and −0.55 eV (SO_2_) for g-ZnCdO_2_. These results suggest chemisorption behavior in most cases, with minimum interaction distances ranging from 2.04 to 2.99 Å. Notably, SO_2_ adsorption on g-ZnCdO_2_ is characterized by a weaker interaction, indicative of weak chemisorption rather than physisorption. Compared to their carbon-based analogues—which often require surface functionalization to enhance gas affinity^11,71,72^ —these inorganic biphenylene and graphenylene frameworks exhibit superior intrinsic adsorption capabilities. Additionally, when compared to the previously reported hexagonal ZnCdO_2_ monolayer,^44^ which was primarily optimized for photocatalytic applications, our b­(g)-ZnCdO_2_ structures demonstrate significantly stronger gas adsorption. By analogy with ZnO-based monolayers, which typically show NO_2_/SO_2_ adsorption energies in the range of −0.4 to −1.0 eV,^73,74^ the much higher adsorption energies observed in our systems (up to −1.88 eV) suggest enhanced gas sensing performance and the potential for efficient room-temperature operation.

**Figure 5:**
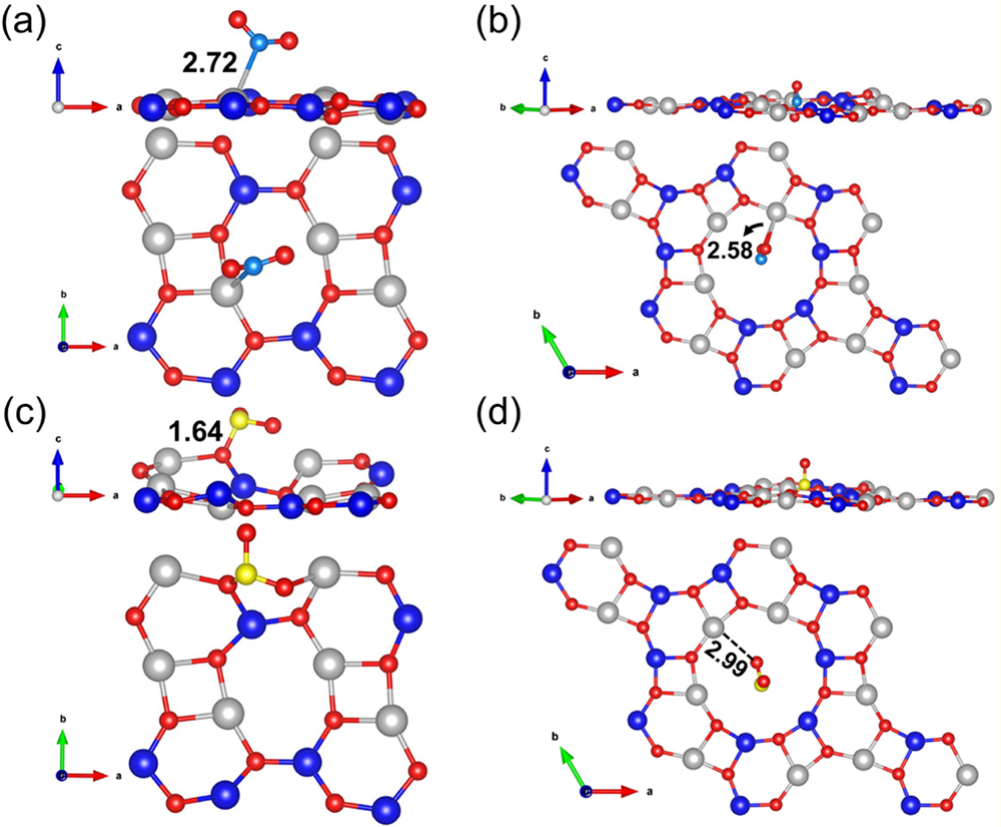
Side and top view configurations of NO_2_ and SO_2_ adsorption on the b-ZnCdO_2_ and g-ZnCdO_2_ monolayers. Panels (a) and (c) show the optimized adsorption geometries on b-ZnCdO_2_, while panels (b) and (d) correspond to g-ZnCdO_2_.

Bader charge analysis further supports this conclusion, revealing notable charge transfer upon adsorption: −0.22*e* and −0.17*e* for NO_2_ and SO_2_ on b-ZnCdO_2_, and −0.44*e* and −0.12*e* on g-ZnCdO_2_, respectively.

The projected density of states was also computed for both NO_2_ and SO_2_ adsorptions on b­(g)-ZnCdO_2_ monolayers, as shown in Figure 6. It can be observed that the introduction of gases alters the electronic response of the studied 2D materials. For example, interaction with NO_2_ increases the band gap of b-ZnCdO_2_ and g-ZnCdO_2_ by 0.09 and 0.03 eV, respectively. In both cases, a significant overlap is observed between the Zn­(d) and Cd­(d) states of the substrate and the O­(p) states from the molecule, indicating a strong binding affinity. On the other hand, SO_2_ causes a slight shift in the pristine b-ZnCdO_2_ band gap, from 1.29 to 1.33 eV, with a notable contribution from the O­(p) orbitals of the gas near the Fermi level (fixed at 0 eV). Although the adsorption magnitude is lower than that of other complexes, the g-ZnCdO_2_+SO_2_ system shows the most significant electronic change, as a new midgap state appears at 1.01 eV (see Figure 6d), with prominent hybridization between the S­(p) orbitals of SO_2_ and the O­(p) states of the g-ZnCdO_2_ monolayer. This midgap state is also observed in other metal oxide semiconductors.^75–77^ Since the practical application of traditional gas sensing devices is closely linked to changes in the material’s conductivity,^78^ we can confirm the high sensitivity of b­(g)-ZnCdO_2_ substrates to NO_2_ and SO_2_ gases.

**Figure 6:**
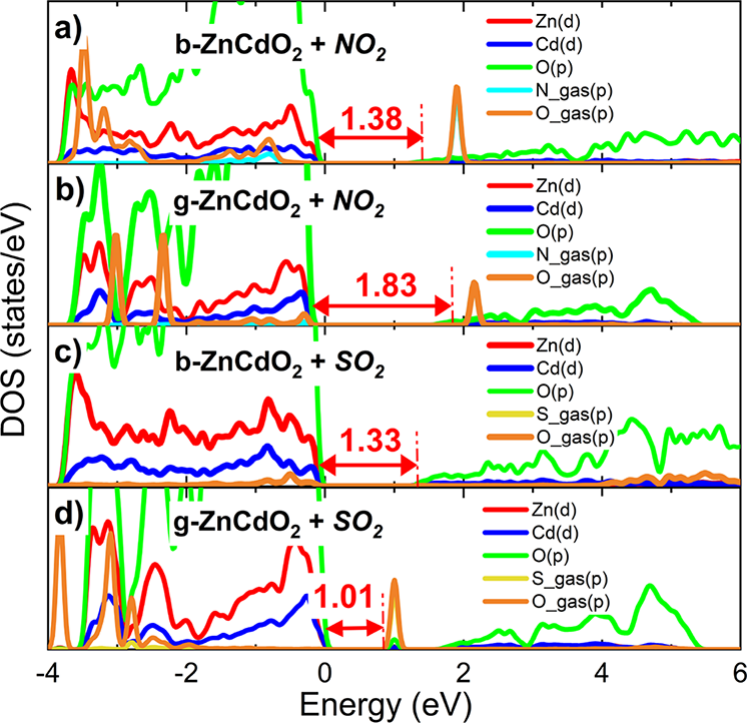
Projected density of states (DOS) for NO_2_ and SO_2_ adsorptions on b-ZnCdO_2_ (a, c) and g-ZnCdO_2_ (b, d) monolayers. Fermi level is fixed at zero eV.

To confirm the previous discussion, this work also investigates the effect of the target gases on potential changes in the work function (WF), as shown in Figure 7. The WF is calculated using the following expression:

$$\phi =E_{\mathrm{vac}}-E_{F}$$ 4

**Figure 7:**
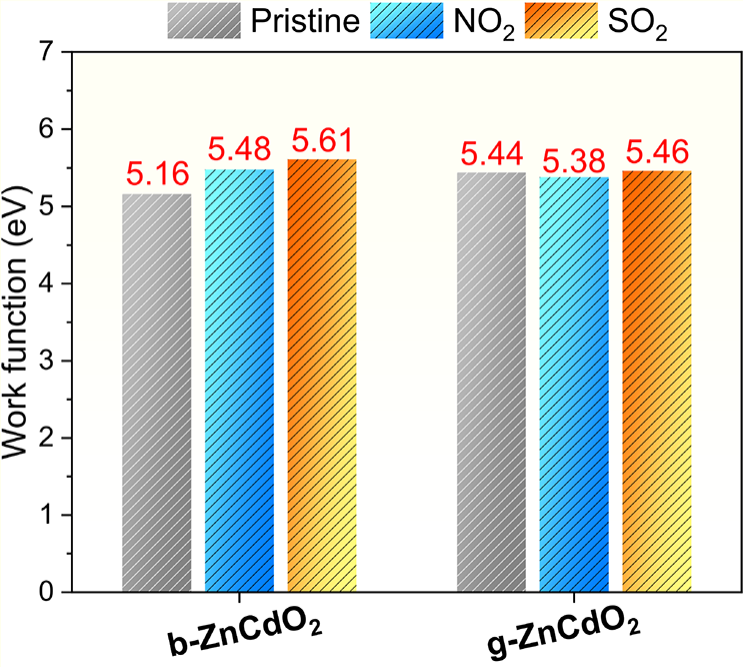
Histogram representation of the work function (WF) values for b-ZnCdO_2_ and g-ZnCdO_2_ monolayers before and after gas adsorption.

where *E*_vac_ and *E*_F_ represent the vacuum potential energy and the Fermi energy, respectively. The Kelvin probe technique is commonly employed to measure these values experimentally, using the work function (WF) to detect adsorbed compounds through variations in the contact potential difference (CPD).^79,80^ For the pristine configuration, the WF of b-ZnCdO_2_ and g-ZnCdO_2_ is calculated to be 5.16 and 5.44 eV, respectively. Upon gas adsorption, notable shifts are observed. In b-ZnCdO_2_, the WF increases by 0.32 eV for NO_2_ and 0.45 eV for SO_2_, indicating strong electronic interactions. Conversely, g-ZnCdO_2_ exhibits smaller changes—0.06 eV (NO_2_) and 0.02 eV (SO_2_)—suggesting comparatively weaker perturbation of its electronic structure. These variations support the presence of chemisorption across all systems, as evidenced by the measurable modulation of the electronic properties upon gas binding.

Finally, the reversibility of the proposed systems was analyzed using the sensor recovery time (τ). This parameter can be derived using transition state theory (TST) and is given by

$$\tau =\nu^{-1}\exp\left(\frac{\left|E_{\mathrm{ads}}\right|}{k_{B}T}\right)$$ 5

where ν is the attempted frequency, *k*_B_ is the Boltzmann constant (8.62 × 10^–5^ eV K^–1^), and *T* is the temperature. The attempted frequency is assumed to be 10^12^ s^–1^, consistent with that of NO_2_.^81^

The recovery time of a gas sensor is linked to the substrate’s ability to be reused. In other words, it reflects the sensor’s practical effectiveness in performing subsequent measurements after initial gas exposure. Therefore, short recovery times indicate good desorption capability, which is associated with a low adsorption magnitude, as shown in eq 5. Conversely, long τ values suggest a challenging scenario for sensor reusability. Table 3 summarizes the results for the NO_2_ and SO_2_ adsorptions on b­(g)-ZnCdO_2_ monolayers as a function of different temperatures (300, 400, and 500 K) and attempted frequencies (visible and UV light). At room temperature, it can be observed that only the g-ZnCdO_2_+SO_2_ system has a short recovery time, calculated to be 1.74 × 10^–3^ and 1.74 × 10^–6^ s under visible and UV light exposure, respectively. However, as the temperature increases, all other systems become suitable for subsequent detection. For example, under visible light at 500 K, the recovery time for NO_2_ on b­(g)-ZnCdO_2_ is τ = 2.69 × 10^4^ s (8.92 × 10^6^ s), which decreases under UV light. A similar trend is observed for SO_2_ on the b-ZnCdO_2_ monolayer, with a desorption time of only 413 s at 500 K.

**Table 3:** Recovery Times for NO_2_ and SO_2_ Detection by b­(g)-ZnCdO_2_ Structures under Visible Light and UV Light Conditions, and Different Temperatures

recovery time (s)			
system	300 K/visible light	400 K/visible light	500 K/visible light
b-ZnCdO_2_/NO_2_	2.42 × 10^15^	3.45 × 10^8^	2.69 × 10^4^
b-ZnCdO_2_/SO_2_	2.29 × 10^12^	1.86 × 10^6^	4.13 × 10^2^
g-ZnCdO_2_/NO_2_	3.84 × 10^19^	4.87 × 10^11^	8.92 × 10^6^
g-ZnCdO_2_/SO_2_	1.74 × 10^–3^	8.51 × 10^–6^	3.50 × 10^–7^

	300 K/UV	400 K/UV	500 K/UV
b-ZnCdO_2_/NO_2_	2.42 × 10^12^	3.45 × 10^5^	26.9
b-ZnCdO_2_/SO_2_	2.29 × 10^9^	1.86 × 10^6^	0.4
g-ZnCdO_2_/NO_2_	3.84 × 10^16^	4.87 × 10^8^	8.92 × 10^3^
g-ZnCdO_2_/SO_2_	1.74 × 10^–6^	8.51 × 10^–9^	3.50 × 10^–10^

While these findings confirm the potential for temperature- and light-assisted recovery, certain systems—namely b-ZnCdO_2_/NO_2_, b-ZnCdO_2_/SO_2_, and g-ZnCdO_2_/NO_2_—exhibit prohibitively long recovery times at ambient conditions. This may limit their practical application in real-time sensing. To address this, material-level optimization is essential. Doping strategies (e.g., with alkali or transition metals) could be used to weaken adsorption strength and accelerate desorption kinetics.^82,83^ Additionally, defect engineering—such as introducing oxygen vacancies or Zn/Cd site substitutions—may enhance charge transfer efficiency and tune surface reactivity, thus improving both sensitivity and recovery behavior.^84,85^ These modifications offer promising pathways for tailoring b­(g)-ZnCdO_2_ monolayers toward practical sensing technologies and merit further investigation.

For broader context, Table 4 compares our results with other widely studied 2D materials for NO_2_ and SO_2_ detection. Compared to conventional 2D sensing materials listed in Table 4, the b­(g)-ZnCdO_2_ monolayers demonstrate superior or competitive performance in several key aspects. The adsorption energies for NO_2_ (up to −1.88 eV) and SO_2_ (−1.45 eV for b-ZnCdO_2_) are notably higher than those of pristine arsenene (−0.44 eV), C_3_N (−0.79 eV), and ZnO (−0.85 eV), indicating stronger gas binding and potentially enhanced sensing sensitivity. Additionally, the associated charge transfers (−0.22 to −0.44*e*) are among the highest reported, rivaling or exceeding that of GeSe (−0.46*e*), which is known for its excellent reactivity. While recovery times for b-ZnCdO_2_ remain relatively long at room temperature, the g-ZnCdO_2_+SO_2_ system shows an exceptionally fast desorption (τ 1.74 × 10^–3^ s), outperforming most existing sensors. These comparisons confirm that the topological design of b­(g)-ZnCdO_2_ offers enhanced interaction strength, improved charge exchange, and selective reusability, positioning these materials as strong candidates for next-generation gas sensors.

**Table 4:** Comparison of Gas Sensing Parameters for Standard 2D Materials

material	gas	*E*_ads_ (eV)	*d*_min_ (Å)	*Q* (*e*)	Δ*E*_g_ (eV)	τ (s)
arsenene^86^	NO_2_	–0.44	2.96	–0.19	0.12	450
arsenene^86^	SO_2_	–0.34	2.96	–0.19	0.09	430
GeSe^87^	NO_2_	–2.24	2.29	–0.46	0.40	30
GeSe^87^	SO_2_	–0.58	2.86	–0.28	0.20	120
borophene^88^	NO_2_	1.75	1.56	0.76	1.12	60
C_3_N^89^	NO_2_	–0.79	2.89	–0.39	0.32	85
C_3_N^89^	SO_2_	–0.62	2.84	–0.25	0.28	160
Blue P^90^	SO_2_	–0.25	3.00	–0.14	0.05	550
Ni–MoS_2_ ^91^	SO_2_	–1.38	2.06	–0.02	0.35	70
GaN^92^	NO_2_	–0.67	2.07	–0.11	0.20	310
ZnO^74^	SO_2_	–0.85	2.45	0.12	0.05	150
CdO^93^	NO_2_	–0.65	2.60	–0.10	0.10	220

## Conclusions

In this study, density functional theory (DFT) simulations were utilized to introduce novel two-dimensional (2D) inorganic ZnCdO_2_ semiconductors with biphenylene and graphenylene lattices, demonstrating their potential for detecting toxic NO_2_ and SO_2_ gases. Phonon dispersion curves confirmed the dynamic stability of both b­(g)-ZnCdO_2_ monolayers. Furthermore, ab initio molecular dynamics (AIMD) simulations at room temperature verified the structural integrity of these materials.

Adsorption analysis revealed strong binding between the b­(g)-ZnCdO_2_ monolayers and the target gases, with most configurations exhibiting chemisorption, as indicated by adsorption energies ranging from −0.55 to −1.88 eV. Notably, the interaction of SO_2_ with g-ZnCdO_2_ is characterized as a case of weak chemisorption, due to its relatively moderate adsorption energy (−0.55 eV), longer interaction distance (2.99 Å), and modest charge transfer (−0.12*e*). Despite this weaker interaction, side-view geometries clearly show SO_2_ binding to the surface, supporting its reversible adsorption behavior. Moreover, gas exposure induces visible changes in the band structure of both monolayers, reflecting high sensor sensitivity. These findings are further validated by shifts in work function (WF) values, confirming the electronic modulation of the monolayers upon NO_2_ and SO_2_ adsorption.

While the predicted sensing performance of the b­(g)-ZnCdO_2_ monolayers is encouraging, several practical challenges must be considered for real-world implementation. Notably, the potential toxicity of cadmium raises environmental and safety concerns, and the synthesis of such topologically complex oxide monolayers may pose fabrication challenges. Additionally, achieving high selectivity under ambient conditions remains a hurdle. These limitations underscore the need for future experimental efforts, including doping and defect engineering strategies, to enhance desorption kinetics, improve material safety, and enable scalable synthesis—ultimately paving the way toward practical gas-sensing technologies.

Altogether, our findings reveal the untapped potential of b­(g)-ZnCdO_2_ monolayers for selective, efficient, and reusable gas sensing, while also outlining clear pathways for experimental realization and performance enhancement in future practical applications.

## Data Availability

All data supporting the findings of this study are available within the article.
